# Parametric analysis of a novel semi-circular microfluidic CD-ELISA valve

**DOI:** 10.1186/1754-1611-5-15

**Published:** 2011-11-07

**Authors:** Samuel I En Lin

**Affiliations:** 1 Department of Power Mechanical Engineering, National Formosa University, Taiwan

**Keywords:** CD-ELISA, microfluidics, valve design, centrifugal force

## Abstract

CD-ELISA uses the microfluidic ranking method and centrifugal force to control the testing solution as it flows into the reaction region. The most challenging part of CD-ELISA is controlling the flow process for different biological testing solutions, i.e. the controlling sequence for the microfluidic channel valves. The microfluidic channel valve is therefore the most important fluid channel structure for CD-ELISA. In this study, we propose a valve design suitable for a wide range rotational speeds which can be applied for mass production (molding). Together with supporting experiments, simulation based on two-phase flow theory is used in this study, and the feasibility of this novel valve design is confirmed. Influencing design factors for the microfluidic channel valves in CD-ELISA are investigated, including various shapes of the arc, distance d, radius r, the location of the center of the circle, and the contact angle. From both the experimental results and the simulated results, it is evident that the narrowest channel width and the contact angle are the primary factors influencing valve burst frequency. These can be used as the main controlling factors during the design.

## 1. Introduction

The current method for biomedical examination tests requires a faster analyzing and determination process. Having the testing samples and testing solutions in smaller scales has become the solution. The principle of ELISA is to show the existence of a particular protein through the specificity of the bond between antigen (i.e. protein) and antibody, demonstrated by a color-change or luminescence reaction with an enzyme [1-3].

Highly specific antibody reactions and magnifying functionality have been widely used in biomedical testing, environmental analysis, and biotechnology research. Conventionally, an antibody with specificity to the expressed protein that targets foreign transformed genes is produced first during the ELISA detection process. This antibody is referred to as the first order antibody (or detection antibody). During the detection process, the body fluid protein to be detected is first placed on a membrane which prevents non-specific antibody binding. The first order antibody is added to detect and capture the specific protein. The second order antibody (or enzyme-labeled antibody) is then added. This second order antibody is linked with an enzyme and has specificity to the first order antibody. The specific linkage produced by the second order antibody and the first order antibody carries the enzyme to the location of the protein, and the enzyme substrate is added. After a period of time, the enzyme catalyzes the substrate causing reactions involving color changes or luminescence. Finally, the existence of the specific protein is confirmed if residual color changes or emissions remain at specific locations on the membrane [3,4].

This conventional method involves excessive numbers of procedures, and each procedure requires separate execution, which significantly lengthens the detection time. CD-ELISA is an ELISA process based on a compact disk. The main principle in this process is to design and construct various microfluidic channels on a blank CD. Test solutions can be placed at specific locations first, and then the test samples can be placed in the center. The CD can then be placed in a reader, and as the CD spins, centrifugal forces are generated, creating the so-called pump. A higher rotational velocity produces a larger centrifugal force, and a lower rotational velocity produces a smaller centrifugal force. This method can be used on samples to first control the direction of flow, and then to create reactions with the testing solutions which are already placed on the CD. The reaction results can be measured via visible or UV light. The measured results are then entered into the computer for analysis.

The amount of testing solution used in CD-ELISA is five times less than that of ELISA. The usage time can also be reduced by as much as nine times [5]. The most challenging aspects of the development process for CD-ELISA are (1) the controlling of flow processes for different biological testing solutions, using microfluidic channel valves to control the sequential orders of the outflow fluid, and (2) the statistics for multi-channel sample detections, using bifurcation technology in fluidic channels to form the splitting effects in multiple microfluidic channels. There have been numerous studies involved with the design of the valve. The valve mechanism can be divided into two categories, namely active and passive. Active valves include diaphragm-type hydraulic control valves [6], surface wetting valves [7], gas bubble valves (controlled by the electrochemical method, [8]), and heat driven gels [9]. With satisfactory designs, micro-pumps can also be used as micro valves. Micro valves based on fluid contact [10], belonging to the passive category, work by placing two capillaries together; for one of them, the fluid stops as soon as it arrives, and for the other one, as the fluid arrives, the fluid changes the equilibrium based on the surface of the static fluid. Recently developed passive valves include the fishbone valve [11] and the well-type valve [12,13]. The fishbone valve is a patented design and is already in commercial usage. However, since the fishbone design has a limited width and is susceptible to breakage, the production process is complicated. The well-type valve design considerably improves this production issue. However, the well-type valve has a small adjustable operational rotation range. When the locations of the valves are fixed, the burst frequency is almost fixed as well. In the overall microfluidic channel design or production process, if there are some errors which require modifications to the valve burst frequency, limitations apply and no modification can be performed.

In this study, we aim for a wide range rotational speed valve for practical usage and propose a novel semi-circular design structure. The effects of various design factors on the burst frequency are studied to find the obvious geometric factors. The Taguchi method is used to further study the effects of geometric factors on the rotational speed. Simulations based on two-phase flow theories are used, with the aid of practical experiments, to confirm the results.

## 2. Two-Phase Flow Theories

Two-phase flow theory is usually used in valve designs [14,3], and in some studies it is used in microfluidic channel design. Two-phase flow theory is necessary because there are large differences between air density and liquid density, and the numeric calculations do not easily converge. In the past, some studies have successfully used two-phase flow theory and incompressible theory in bifurcation design for microfluidic channels [15]. For the same structure, calculated results using both theories have minimal or no difference. In this study, the Cahn-Hilliard equations were used in two-phase flow theory for simulation [16,17]. The intersection of the two-phase interfaces is defined as the phase field variable *ϕ *which varies between -1 and 1. The Cahn-Hilliard equations are first divided into the following equations:

(1) $$\frac{\partial u}{\partial t}+u\cdot \nabla \phi =\nabla \cdot \frac{\gamma \lambda }{\in^{2}}\nabla \psi$$

(2) $$\psi =-\nabla \cdot \in^{2}\nabla \phi +\left(\phi^{2}-1\right)\phi$$

where **u**, γ, λ, and *ε *represent flow velocity of the fluid, liquidity, mixing energy density, and interface thickness parameter, respectively, and *ψ *is the phase field helper variable. Generally, $\lambda =\frac{h_{c}}{2}$ where *h_c _*depends on the size of the feature mesh on the interface, and γ = *ε*^2^.

When applied in terms of the phase field, the volumes for the two fluids are represented separately as below:

(3) $$V_{f1}=\frac{1-\phi }{2}$$

(4) $$V_{f2}=\frac{1+\phi }{2}$$

In this study, liquid 1 is defined as water and liquid 2 is defined as air. The viscosity (*η*) and the density (*ρ*) over the mixing region are defined as:

(5) $$\rho =\rho_{w}+\left(\rho_{a}-\rho_{w}\right)V_{f2}$$

(6) $$\eta =\eta_{w}+\left(\eta_{a}-\eta_{w}\right)V_{f2}$$

where the subscript *w *is for water and the subscript *a *is for air. The transfer functions for momentum and mass are derived from Navier-Stokes equations, expressed as below.

(7) $$\rho \left(\frac{\partial u}{\partial t}+u\cdot \nabla u\right)=-\nabla p+F+F_{st}+\mu \nabla^{2}u$$

(8) ∇⋅u=0

where *ρ*, **u**, p, *μ*, **F**, and **F***_st _*are the density, velocity, pressure, body force, and surface tension of the fluids, respectively. The surface tension **F**_st _can be represented as

(9) $$F_{st}=\nabla \cdot T=\nabla \cdot \left[\sigma \left\{I+\left(-nn^{T}\right)\right\}\delta \right]$$

where *σ *is the surface tension coefficient. At room temperature, the surface tension coefficient for water and air is approximately *σ *= 0.0741 $\frac{N}{m}$. Supposing | is the identity matrix **n **is the unit normal of the surface, and **± **is the Dirac delta function, the body force (**F**) can be represented as:

(10) $$F=\rho_{1}g+F_{ce}+F_{co}=\rho_{1}\left(g+|\omega {|}^{2}r-2\omega \times u\right)$$

where **g**, **r**, and *ω *represent gravity, radius, and angular velocity, respectively. In 3D simulation, since the fluid requires taking the gravity into consideration, the body force represented by vectors therefore contains one additional term, gravity **F**_z_. Further representation of the velocity field is represented as:

(11) $$u=u_{x}i+u_{y}j+u_{z}k$$

And the angular velocity and gravity are represented as:

(12) $$\omega =\omega_{z}k,\;g=g_{z}k$$

Regarding boundary conditions, the wetted wall is defined as one of the boundary parameters and the contact angle is set to be *θ*_**w **_= 70°. Since this is a 3D simulation, the parameter for the surrounding channel wall is applied in the same manner. Fluid is driven by rotation in this study, and therefore motion is generated from centrifugal force due to rotation. As a result, initial conditions for equation (7) are not required.

The contact interface between water and air is configured as the initial fluid interface.

(13) $$\left[-pI+\mu \left(\nabla u+{\left(\nabla u\right)}^{T}\right)\right]n=0$$

Note that, generally, angular acceleration is included as one of the entries for body force, however during the actual execution of the experiment, changes in the rotation speed (e.g. 1000 to 1500 rpm) can be achieved within two seconds, and thus the angular acceleration is neglected. The angular acceleration is also neglected during the process for solving both theories.

## 3. Simulation of valve structure parameters

**Figure 1:**
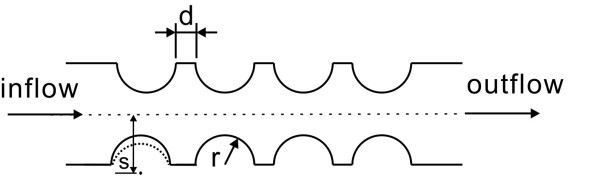
**Illustration of the semi-circular microfluidic channel valve structure**.

The proposed structure in this study is illustrated in Figure 1, where the geometric parameters r, s, and d are shown. The range of rotation speeds is set at 1300~1500 rpm. The effect of the Coriolis force is not noticeable when the rotation speed is very low (i.e. less than 500 rpm). Therefore, low speed rotation is not included for consideration in this study [18]. Even though this has no effect on the valve design, it has significant effects on other devices (e.g. bifurcation point). In terms of the overall CD-ELISA design, it is not practical to have a rotational speed less than 500 rpm. The parameters used during simulation are listed in Table 1. The width of the microfluidic channel is selected to be 300 micrometers, identical to previous experiments from earlier publications [15,3]. Before we investigate the new valve structure, we first perform analysis on rotational speed for fish-bone valve structures.

**Table 1:** Working parameters used in the two-phase flow analysis of the valve design

Parameters	values
Density(water)	997 kg/m^3^

Dynamic viscosity(water)	9.81 × 10^-4 ^N/m^2^

Density(air)	1.293 kg/m^3^

Dynamic viscosity(air)	1.71 × 10^-5 ^N/m^2^

Rotation (RPM)	1300

Channel width and height (mm)	0.3 by 0.3

Gravity	9.8 N/m^2^

Surface tension coefficient	0.0741 N/m^2^

Contact angle	Pi/2.4

Distance to center	26 mm

### 3.1 Analysis of the parametric design for fish-bone valves

The burst frequency of the n^th ^fishbone is a function of twelve parameters: the air/liquid surface tension (z_1_), the contact angles of the liquid from the top view (z_2_), the top contact angle of the liquid from the side view (z_3_), the bottom contact angle of the liquid from the side view (z_4_), the density of the fluid (z_5_), the distance between the center of the CD and the beginning of the fluid in the reservoir (z_6_), the distance between the center of the CD and the end of the fluid flow front (z_7_), the width of the channel (z_8_), the height of the channel (z_9_), the width of a fishbone (z_10_), the distance between fishbones (z_11_), and the number of the fishbone (z_12_) within the fishbone valve. The burst frequency, *f_bn_*, can be expressed in mathematical terms [19].

(14) $$f_{bn}=\sqrt{\left(\frac{z_{1}}{4\pi^{2}z_{5}\left[z_{7}-z_{6}+\left(z_{12}-1\right)\left(z_{11}+z_{10}\right)\right]\left[\frac{z_{6}+z_{7}+\left(z_{12}-1\right)\left(z_{11}+z_{10}\right)}{2}\right]}\right)}\cdot \sqrt{\left(\frac{2\cdot \sin\left(z_{2}\right)}{z_{2}}-\frac{\cos\left(z_{3}\right)}{z_{9}}-\frac{\cos\left(z_{4}\right)}{z_{9}}\right)}$$

**Figure 2:**
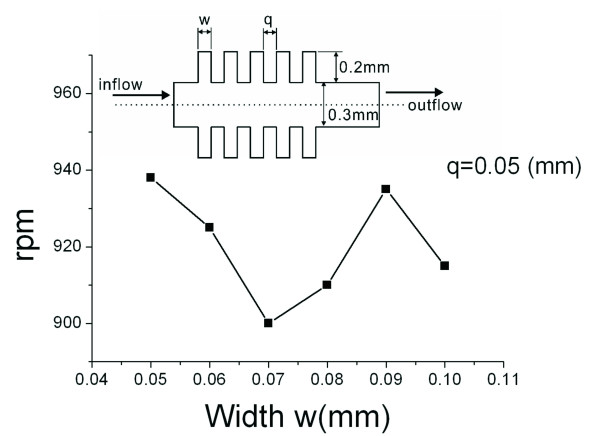
**Stream lines for width w and rotational speed rpm**.

Once the fluid, packaging process, location, and valve surface modification for CD-ELISA have been confirmed, these 12 factors basically reduce to three (z_10_, z_11_, and z_12_). In equation 14, compared with z_10 _and z_11_, the parameter z_12 _(number of the fishbone) has less influence on the burst frequency. In practical usage, z_12 _is used to extend the time for fluid to pass through the valve, and it does not change the burst frequency [19]. We can therefore fix the value for this parameter, and discuss the other two parameters which are more influential. The model we use is a fish-bone valve structure (with z_12 _= 5), as illustrated in Figure 2. There are two variable parameters: the width w for the valve can be varied from 50 and 100 um, and the distance q from 60 to 100 um. In this study, the depth for all of the microfluidic channels is 300 micrometers, the direction of rotation is clockwise, the rotational speed is 800 to 1000 rpm, and the simulation time is 0.1 sec, until the fluid flows out. As shown in Figure 2, as the width w changes, burst frequencies do not express any patterns, and range variation is in a small range.

**Figure 3:**
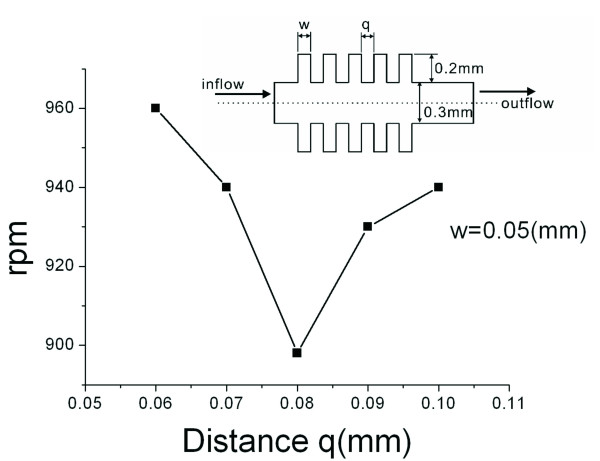
**Stream lines for distance q and rotational speed rpm**.

Figure 3 illustrates the effect of distance q on the burst frequency. It is observed that as the distance (q) increases, changes in rpm do not express any patterns. These results agree with other studies [19]. Based on these results, this type of structure cannot achieve control over a wide range of rotational speeds. Despite the fact that we changed the width and the distance between the teeth, the resulting burst frequency has limited range, and no definite pattern is observed. Hence, we modify the valve structure from fish-bone to semi-circular to perform further investigations.

### 3.2 Analysis of the parametric design for semi-circular valves

**Figure 4:**
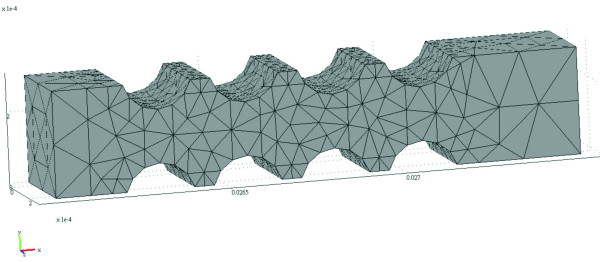
**Illustration of the mesh in the semi-circular microfluidic channel valve**.

**Figure 5:**
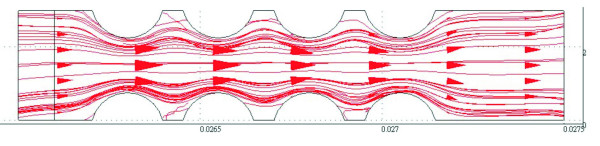
**Simulated stream lines and arrows for the semi-circular microfluidic channel**.

The model we use is a semi-circular valve structure, as illustrated in Figure 4. A total of 5088 elements are used in the 3D model. Since the entire valve does not have a lean and narrow micro-structure, eruption is unlikely to occur during the process of molding/ejection and, which allows a high yield rate during mass production. In practice, when the depth and the width of the microfluidic channel are fixed, there are three variable parameters (shown in Figure 1): (1) radius r can be varied from 0.08 mm to 0.12 mm; (2) distance d from 0.06 mm to 0.1 mm; and (3) distance s from 0.155 mm to 0.175 mm. Distance d is the distance between two semicircles, and distance s is the distance between the center of the circle and the center line. In this study, the depth for all of the microfluidic channels is 300 micrometers, the direction of rotation is clockwise, the rotational speed is varied around 1000 rpm, and the simulation time is 0.1 sec. Similar to the fish-bone valve principle, the ability of the fluid to pass through depends on the centrifugal force and the surface tension of the liquid. As soon as the valve opens, the flow speed as the fluid passes through the narrow channels in the valve would increase, and the stream lines are illustrated in Figure 5. The size of the cross-section affects the flow speed. Compared with the bifurcation design [15], the effect of Coriolis forces is small, and the changes on the cross section are not dramatic; therefore the changes in flow speed are very smooth.

**Figure 6:**
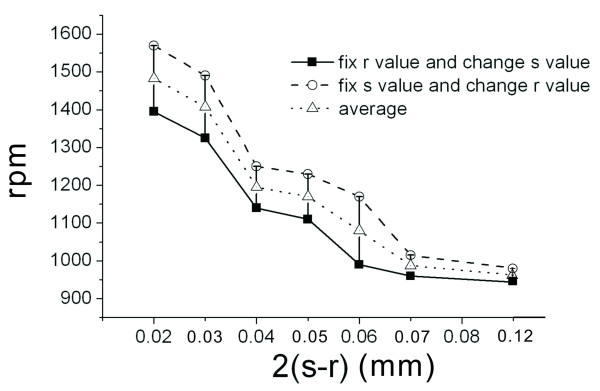
**Stream lines for the narrowest value of the microfluidic channel (i.e. 2x(s-r)) for the semi-circular microfluidic channel and the rotational speed (rpm)**.

**Figure 7:**
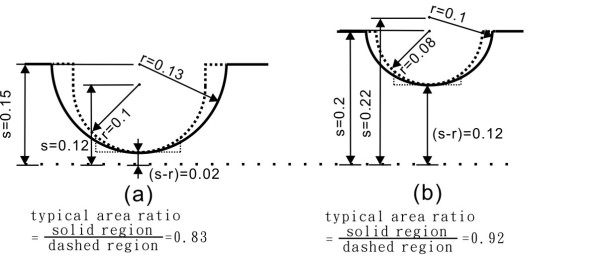
**Various structures**. (a) Two possible structures when the narrowest width for the microfluidic channel is 0.04 mm. One structure has a fixed radius r and varying s (dotted line). The other structure has a fixed radius s, and varying r (solid line). (b) Two possible structures when the narrowest width for the microfluidic channel is 0.240 mm. One structure has a fixed radius r and varying s (dotted line). The other structure has a fixed radius s, and varying r (solid line). The marked units are in mm.

After fixing two parameters (d at 0.05 mm and s at 0.15 mm), we investigate the effects of changes in radius r on the burst frequency. Simulated results are shown in Figure 6. It is observed that larger values for radius r (or smaller values for s-r) cause larger values in burst frequency (rpm). When the radius r is between 0.11 and 0.13 mm (or when the s-r value is between 0.02 and 0.04 mm), the changes are most noticeable, and the average rotational speed increases from 1220 to 1520 rpm. The burst frequency when the narrowest width of the microfluidic channel is 40 um (or s-r is 0.02 mm) can differ from the burst frequency when the narrowest width for the microfluidic channel is 240 um (or s-r is 0.12 mm) by as much as 500 rpm. This is very useful in practical application. Figure 7 illustrates the reasons for having different burst frequencies when the width of the microfluidic channel is the same. Figure 7(a) shows the two possible structures when the narrowest width for the microfluidic channel is 40 um. One structure has a fixed radius r and variable s (dotted line), and the other structure has a fixed radius s, and variable r (solid line). These two structures cover different contacting surface areas of the microfluidic channel under the same radius region (in this sample structure, the ratio for these two areas is approximately 0.83). The angle between the structure and the direction of flow are different for these two structures, and therefore the resistance encountered by the fluid is different. For the first (dotted line) structure, the resistance is greater. Additionally, the contacting surface area is greater than the second (solid line) structure. The burst frequency would be higher than the second structure (about 200 rpm, as shown in Figure 6). Figure 7(b) shows the two possible structures when the narrowest width for the microfluidic channel is 240 um. In this case, since the microfluidic channel is much wider, the regions covered by both structures, the dotted line or solid line, are similar to each other (in this sample structure, the ratio for these two areas is approximately 0.92). The effect of fluid resistance decreases since the microfluidic channel is wider. The resultant burst frequencies by both structures show little difference (about 50 rpm, as shown in Figure 6).

**Figure 8:**
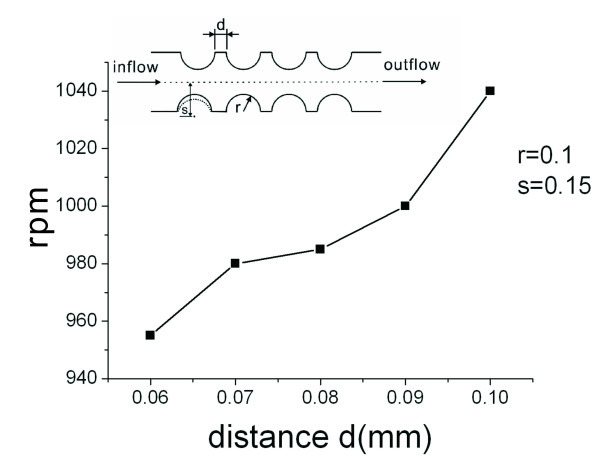
**Stream lines for distance d and rotational speed rpm for the semi-circular microfluidic channel**.

We then investigate the effects of distance d on the burst frequency. In this case, we fix the parameters and set the radius r to be 0.1 mm and the distance s to be 0.15 mm. The simulated results are shown in Figure 8. From the figure, it can be observed that the distance d is proportional to the burst frequency. After comparing the results with Figure 6, it is observed that changes in the burst frequency are relatively smaller. When the narrowest width for the microfluidic channel (s-r) is between 0.06 mm and 0.13 mm, no obvious changes are noted in the burst frequency (Figure 6). We employ the Taguchi method to study the interaction effect of these three geometric parameters on the burst frequency in this region of small changes.

### 3.3 Parametric design for the Taguchi Method

To obtain an optimal design, we first analyse the properties of the key parameters to determine the factors and the number of levels, based on the Taguchi quality engineering method. After considering the extent of interactive effects, we then choose the appropriate orthogonal arrays to perform factor allocation and use it as a reference for system design and execution in the future. In the process of parametric design, we aim to determine the optimum factor level combination, and the selection is based on the value of the S/N ratio. In each factor, there is always one with the largest S/N ratio. This is the optimal level for this factor. Through this analysis, we can control the behavior of the factor around the region of this level. A large S/N value indicates a small change in quality, which means it is closer to the ideal goal.

Based on the analysis results in the previous section, we select the design factors and decide the levels for each factor, as shown in Table 2. The corresponding orthogonal arrays *L*_9 _(3^3^) are constructed as shown in Table 3. Experiments involving many factors can be done all at once. Using Table 3, each influential factor in the orthogonal table can be searched to find the effective method within a limited number of experiments.

**Table 2:** Values of levels for each controlling factor

Geometric Parameter	R	D	S
**Factor**	**A**	**B**	**C**

Leve1 1	0.08	0.06	0.155

Leve1 2	0.1	0.08	0.165

Leve1 3	0.12	0.1	0.175

**Table 3:** *L*_9 _(3^3^)Orthogonal Arrays and the burst frequency obtained by simulation

	R	D	S	RPM
**1**	1	1	1	975

**2**	1	2	2	1035

**3**	1	3	3	978

**4**	2	1	2	900

**5**	2	2	3	890

**6**	2	3	1	1020

**7**	3	1	3	1000

**8**	3	2	1	1260

**9**	3	3	2	1090

**Figure 9:**
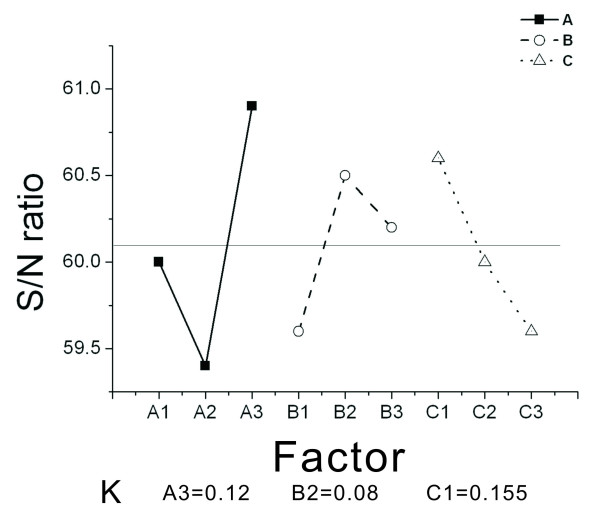
**Corresponding diagram for the Larger The Better (LTB) characteristic**.

**Figure 10:**
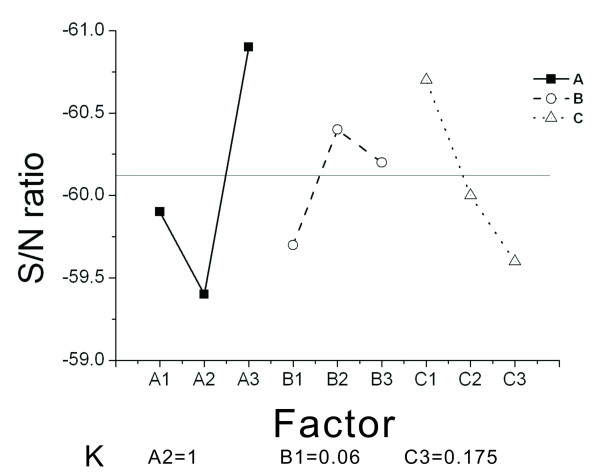
**Corresponding diagram for the Smaller The Better (STB) characteristic**.

Based on this selection principle, the values which optimize the design are selected, and the minimum and maximum average S/N ratio for each factor level is searched. Figure 9 shows the corresponding factor diagram for the 'Larger The Better' (LTB) characteristic. For the level of optimal LTB factors, the combination is A3 = 0.12, B2 = 0.08, and C1 = 0.155. Figure 10 shows the corresponding factor diagram for the 'Smaller The Better' (STB) characteristic. For the level of optimal STB factors, the combination is A2 = 1, B1 = 0.06, and C1 = 0.175. The optimal combination can be obtained from the eighth combination in orthogonal arrays *L*_9 _(3^3^) in Table 3, which indicates a higher rotational speed.

For the LTB characteristic in Figure 9, it is observed that the average value is 0.61 and only A3 is greater than the average value. In Table 2, for the corresponding A3 with the value of 0.12 mm, B2 and B3 are both greater than the average value. Since B2 is greater than B3, B2 is used. In Table 2, for the corresponding B2 with a value of 0.08 mm, only C1 is greater than the average value. In Table 2, the corresponding C1 has a value of 0.155 mm.

The value of K can be calculated as *k *= *S*/*N_max _*- *S/N_min _*where K is the variance of the factors in the experiment, or the difference between the maximum value and the minimum value of the S/N ratio. A larger K value means a larger variance, which shows that this factor has a greater influence.

Figure 9 shows that the K value for factor A is 1.5, the K value for factor B is 0.9, and the K value for factor C is 1. Since the K value for factor A is larger, a larger radius r causes greater changes in rpm.

For the STB characteristic in Figure 10, it is observed that the average value is -60.1. A2 is smaller than the average value. In Table 2, for the corresponding A2 with a value of 0.08 mm, B1 is smaller than the average value. In Table 2, for the corresponding B1 with a value of 0.06 mm, C2 and C3 are smaller than the average value. Since C3 is smaller than C2, C3 is used. In Table 2, the corresponding C3 has a value of 0.175 mm. Figure 10 also shows that the K value for factor A is 1.5, the K value for factor B is 0.7, and the K value for factor C is 1.1. Since the K value for factor A is relatively larger comparing both LTB and STB characteristics, factor A has a greater effect on burst frequency.

## 4. Experimental verification

### 4.1 Experimental setup

Polymethyl methacrylate (PMMA) CD-like plates were used in the experimental study. Microfluidic channels were machined using high-speed (50 K RPM) Computer Numerical Control (CNC) machining on the flat PMMA channel surface. A total of four valves are applied in practice (Table 4). Due to the limitation in tooling size in practice, the narrowest width for the valve is only 260 um. The value for the distance parameter, s, is therefore larger than the configured values in Table 2. During this study, in order to eliminate effects other than the Coriolis force, the inflow channels (before each valve in the PMMA CD test bench) were aligned in a radial direction. De-ionized (DI) water was added by using a volume control pipette and was applied in the loading area each time. There were 40 runs in each group and measurements on the burst frequency (i.e. rpm) were carried out accordingly. A CCD image capturing facility is used in real time to examine whether the fluid passes through the valve opening. The motor was connected to an encoder to trigger a strobe (Monarch, DA 115/Nova Strobe) for synchronized imaging. The loading area was designed to be smaller than the detection area so that there would not be any issues of overflowing during experiments. The ELISA packaging is the same as previously published work [15] and will not be described here in detail.

**Table 4:** Geometric parameters for four valves in the practical experiment

Model #	R(mm)	D(mm)	S(mm)
**1**	0.08	0.06	0.205

**2**	0.1	0.08	0.225

**3**	0.12	0.08	0.205

**4**	0.12	0.1	0.215

The CD-based microfluidic platform with a programmable stepping motor was also utilized in this experiment. The computer-controlled spindle can run at a constant rotational speed for a specified time period and move to the next specified speed. All channels were cleaned and dried using an antistatic air-gun each time in order to have uniform surface quality across all experiments. The channel surface quality (i.e. contact angle) can affect the flow velocity and therefore plays an important role in the burst frequency [15]. The general channel roughness of a molded PMMA CD ELISA is around Ra = 0.34~0.43 micrometers [15].

### 4.2 Experimental verification

**Figure 11:**
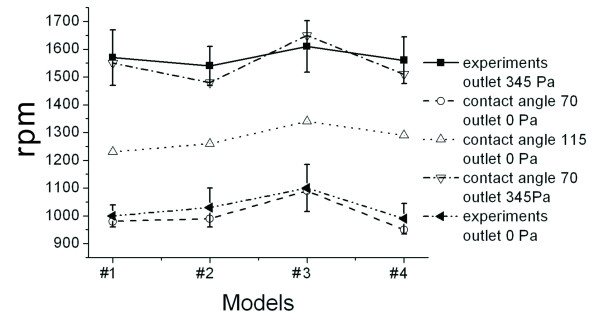
**Comparison of the experimental data and the simulated results**. If there is an air outlet, then the pressure at the inlet would equal the pressure at the outlet.

In Figure 11, the simulated data is compared with the experimental data. For the experiment, if the CD does not have a reserved air-outlet (opening) after the packaging process, the inlet pressure and the outlet pressure would be different since fluid compresses air. In simulation, this factor should be considered. In the case where there is no reserved air-outlet (or there is no connection between the inlet and the outlet), the burst frequency would increase. In terms of the current valve geometry in the experiment, the burst frequency would increase from 1000 to 1500 rpm. The contact angle also directly affects the burst frequency. A larger contact angle causes a higher burst frequency. When the contact angle increases from 70 degrees to 115 degrees, the burst frequency also increases from 1000 to 1300 rpm. After processing, the contact angle for the current valves is approximately 70 degrees [15], therefore the experimental data agrees with the simulated results. For the current practical valve geometry, since the narrowest value for the width of the microfluidic channel is 260 nm, the adjustable range for burst frequency is relatively smaller, within the range of 100 to 200 rpm.

## 5. Conclusions

There are many geometric parameters which affect the burst frequency of the microfluidic channel. In practical application, we must consider the yield rate in processing (related to production cost), wide range of rotational speeds (feasibility of the CD size), sensitivity of each parameter to rotational speed (design control), etc. Further investigation of the geometric parameters for the valve is essential. Combining simulated results with experimental verification, it is confirmed that the valve design structure for microfluidic channels proposed in this study can be applied in CD-ELISA. The following conclusions are obtained:

1. From the simulated results, it can be observed that using an appropriate selection of parameters, this valve design can cover 500 rpm in terms of the burst frequency range. This is approximately 5 times more than the conventional fish-bone valve (about 100 rpm). This offers a more flexible design option for the location of the valve on the CD spin disk. In addition, since the design does not contain microstructures, it is unlikely to erupt, which offers a more stable yield rate in production.

2. Comparing the three parameters, r, s, and d, used in the semi-circular valve structure, when d is fixed, the narrowest width for microfluidic channel (i.e. 2 times the s-r value) is most influential on burst frequency. In terms of the geometry used in this study, we can use the microfluidic channel width to control the burst frequency when the width is smaller than 140 um. The adjustable range can be 500 rpm. For the same microfluidic channel width, the size structure of the radius can also change the burst frequency (about 200 rpm). For microfluidic channel widths greater than 140 um, the aid of surface modification is required, and the contact angle needs to be adjusted. During production control, it is particularly important to emphasize controlling the narrowest microfluidic channel width.

3. In practical experiment verification, the magnitude of the contact angle, the pressure at the inlet, and the pressure at the outlet all directly affect the burst frequency. The simulated results and the experimental data agree with each other.

## 6. Competing interests

The authors declare that they have no competing interests.
